# Single-layer phase gradient mmWave metasurface for incident angle independent focusing

**DOI:** 10.1038/s41598-021-92083-5

**Published:** 2021-06-16

**Authors:** Wonwoo Lee, Semin Jo, Kanghyeok Lee, Hong Soo Park, Junhyuk Yang, Ha Young Hong, Changkun Park, Sun K. Hong, Hojin Lee

**Affiliations:** 1 Department of Information Communication Convergence Technology, Soongsil University, Seoul, 0678 South Korea; 2 School of Electronic Engineering, Soongsil University, Seoul, 0678 South Korea

**Keywords:** Energy science and technology, Engineering, Optics and photonics

## Abstract

Metasurfaces allow the rapid development of compact and flat electromagnetic devices owing to their capability in manipulating the wavefront of electromagnetic waves. Particularly, with respect to the metasurface lenses, wide operational bandwidth and wide incident angle behavior are critically required for practical applications. Herein, a single-layer phase gradient metasurface lens is presented to achieve millimeter-wave focusing at a focal point of 13 mm regardless of the incident angle. The proposed metasurface lens is fabricated by constructing subwavelength-thick (< λ/10) phase elements composed of two metallic layers separated by a single dielectric substrate that exhibits low-Q resonance properties and a wide phase modulation range with satisfactory transmissivity. By controlling the spatial phase distribution, the proposed metasurface lens successfully realises effective wavefront manipulation properties and high-performance electromagnetic-wave-focusing characteristics over a wide operating frequency range from 35 to 40 GHz with incident angle independency up to 30°.

## Introduction

With the advent of metamaterials, artificially engineered materials that enable the manipulation of electromagnetic waves, i.e., two-dimensional planar metamaterials referred to as metasurfaces, are garnering significant attention^1–5^. Owing to their capability in manipulating the phase, amplitude, and polarisation of electromagnetic waves, metasurfaces allow the rapid development of compact and flat electromagnetic devices for recent integration-optics applications^6–9^. Using carefully devised metasurfaces, unique unprecedented electromagnetic properties have been achieved, such as anomalous reflection and refraction^10–13^, planar lens^14–17^, plasmonic lithography^18,19^, and computational holographic imaging^20–22^. In particular, metasurface lenses or meta-lenses can operate as flat lenses for converting an incident plane wavefront to a spherical wavefront with tailored hyperbolic phase distributions on the metasurface. Owing to their low profile, low loss, and ease of fabrication, metasurface lenses have become indispensable elements for planar optical devices, whereas conventional optical devices generally rely on gradual phase accumulation in bulk materials. Hence, phase gradient metasurfaces have been highlighted as a promising candidate for realising electromagnetic-wave-focusing characteristics by manipulating the wavefront through controlling the spatial phase and transmission profiles of metasurfaces.

Phase gradient metasurfaces were first demonstrated by Yu et al. in 2011 using nano-V-antennas of different shapes to verify the generalised Snell’s laws^23^, ranging from microwave^24–26^ to visible regimes^27–29^. Typically, the key characteristics of a metasurface lens include wide phase-shift coverage range, operating frequency range, and incident angle dependency. In particular, wide phase modulation with high transmission efficiency is required to effectively and accurately manipulate the incident wavefront; however, it is difficult to generate a full phase range from 0 to 2$\user2{\pi }$ using only a single-layered structure. Therefore, the stacking of multiple metasurface layers has been suggested to achieve high-performance metasurface lenses^30,31^, phase-shifting surfaces^32^, and transmit-array antennas^33,34^ recently. However, despite their favourable characteristics, multilayer stacking methodologies are not an optimal solution for developing compact and lightweight microwave devices for mobile applications.

Furthermore, it is critical for the lenses to maintain the focusing features, including focal point, focal length, and focused field intensity over a wide range of incident angles. Therefore, numerous approaches have been proposed to impose incident angle independency on designated lenses or metasurfaces^35–37^. However, the majority of the methodologies developed hitherto have been demonstrated only in the visible regime, and analytical studies in the millimeter-wave frequency range are scarce.

Herein, we propose a single-layer phase-gradient metasurface lens with a subwavelength thickness that is capable of effectively controlling the spatial phase and transmission distribution with low-Q resonance properties, as well as with incident angle independency over a wide operating frequency band. From the experimental results, we confirm that the proposed metasurface exhibits electromagnetic-wave-focusing characteristics from 35 to 40 GHz and maintains a spatially fixed focal point at 13 mm for incident angles from − 30° to 30°.

## Results

**Figure 1:**
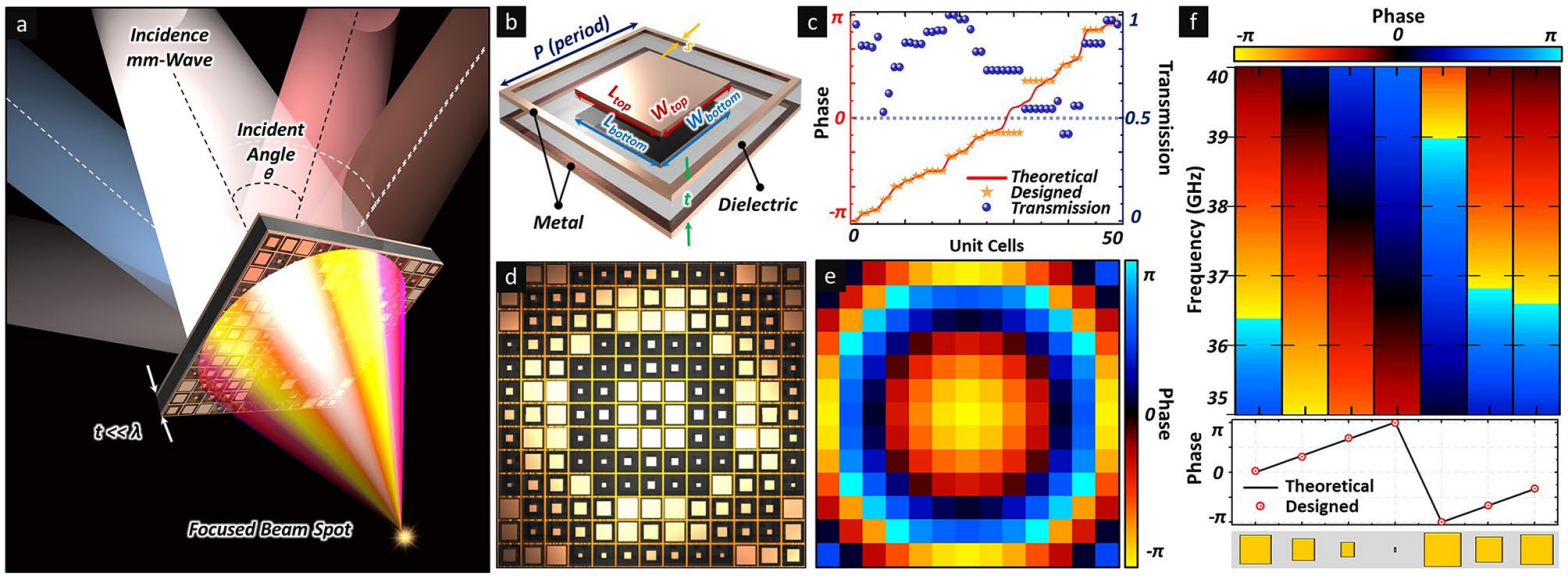
(**a**) Schematic illustration of electromagnetic wave focusing characteristics of proposed phase gradient single-layer metasurface lens. (**b**) Structure of unit cell composed of two metallic layers separated by single dielectric substrate. (**c**) Simulated and experimental phase and magnitude responses of designed unit cells. (**d**) Layout of phase gradient metasurface lens. (**e**) Theoretical phase distribution of proposed metasurface lens. (**f**) Simulated broadband phase gradient spectrum (top) and phase responses of each structure (bottom).

A schematic illustration of the electromagnetic-wave-focusing performance of the proposed phase gradient single-layer metasurface lens is illustrated in Fig. 1a. The subwavelength thickness of the unit cell array was considered to realise broadband operating and incident-angle-independent characteristics. The structure of the unit cell, as shown in Fig. 1b, comprised a single dielectric layer sandwiched between two metallic films. Each metallic layer comprised an outer square ring and inner solid square patch, as shown in Fig. 1b, where *p* (= 3 mm) is the period of the unit cells, *t* (= 0.787 mm) is the thickness of the dielectric substrate, *s* (= 0.1 mm) is the line width of the outer square rings, and *W*_*top,bottom*_ and *L*_*top,bottom*_ are the width and length of the solid square patch for the top and bottom layers, respectively. The dielectric layer had a permittivity of $\varepsilon _{r}$ = 2.2 and a loss tangent of $tan{}\delta$ = 0.0009.

To focus the incident wave, the discrete phase distribution on the phase gradient metasurface should satisfy the hyperbolic formation as given by Eq. (1) owing to Fermet’s principle^38^, where $\user2{\varphi }_{\user2{r}}$ is the phase difference of unit cells, $\lambda$ is the free-space wavelength, $\user2{r} = ~\sqrt {\user2{x}^{2} + \user2{y}^{2} }$ is the distance between the origin (*x* = 0, *y* = 0) and point (*x*, *y*) on the metasurface, and f is the focal length.

1 $$\varphi _{r} = {}\frac{{2\pi }}{\lambda }\left( {\sqrt {r^{2} + f^{2} } - f} \right).$$

In general, to effectively manipulate the wavefront of electromagnetic waves, full-range phase control with high efficiency is desired. In this regard, the phase modulation range and transmission coefficient for the proposed unit cells were investigated using a High-Frequency Structure Simulator (HFSS), as shown in Fig. 1c. By manipulating geometric parameters *W*_*top,bottom*_ and *L*_*top,bottom*_, a wide phase modulation range and an average transmission coefficient exceeding 0.75 were achieved to obtain the full control of phase shift from 0 to 2$\user2{\pi }$. Based on the optimised phase elements, the single-layer electromagnetic-wave-focusing metasurface was realised by arranging the unit cells in a 13 × 13 array measuring 39 mm × 39 mm, as shown in Fig. 1d (see Supplementary Fig. S1). Based on the theoretical calculation using Eq. (1) for a specific frequency and focal length, the phase distribution on the metasurface can be determined, as shown in Fig. 1e. Unit cells with various corresponding geometric parameters were implemented and demonstrated good agreement with the theoretical phase values at the design frequency of 38 GHz. Furthermore, to estimate the operating frequency range of the designed unit cells, the spectral transmission phase responses of specific phase elements with different geometric parameters were investigated across a frequency range from 35 to 40 GHz, as shown in Fig. 1f. As illustrated in the figure, the full range phase modulation from 0 to 2$\user2{\pi }$ was achieved across the desired frequency range by changing the geometric parameters of the unit cell. This result can be attributed to the arrangement of the low-Q resonance property of unit cells throughout a broad frequency range (see Supplementary Fig. S2), and the feasibility of effective wavefront engineering by controlling the phase distributions on the proposed single-layer metasurface was confirmed.

**Figure 2:**
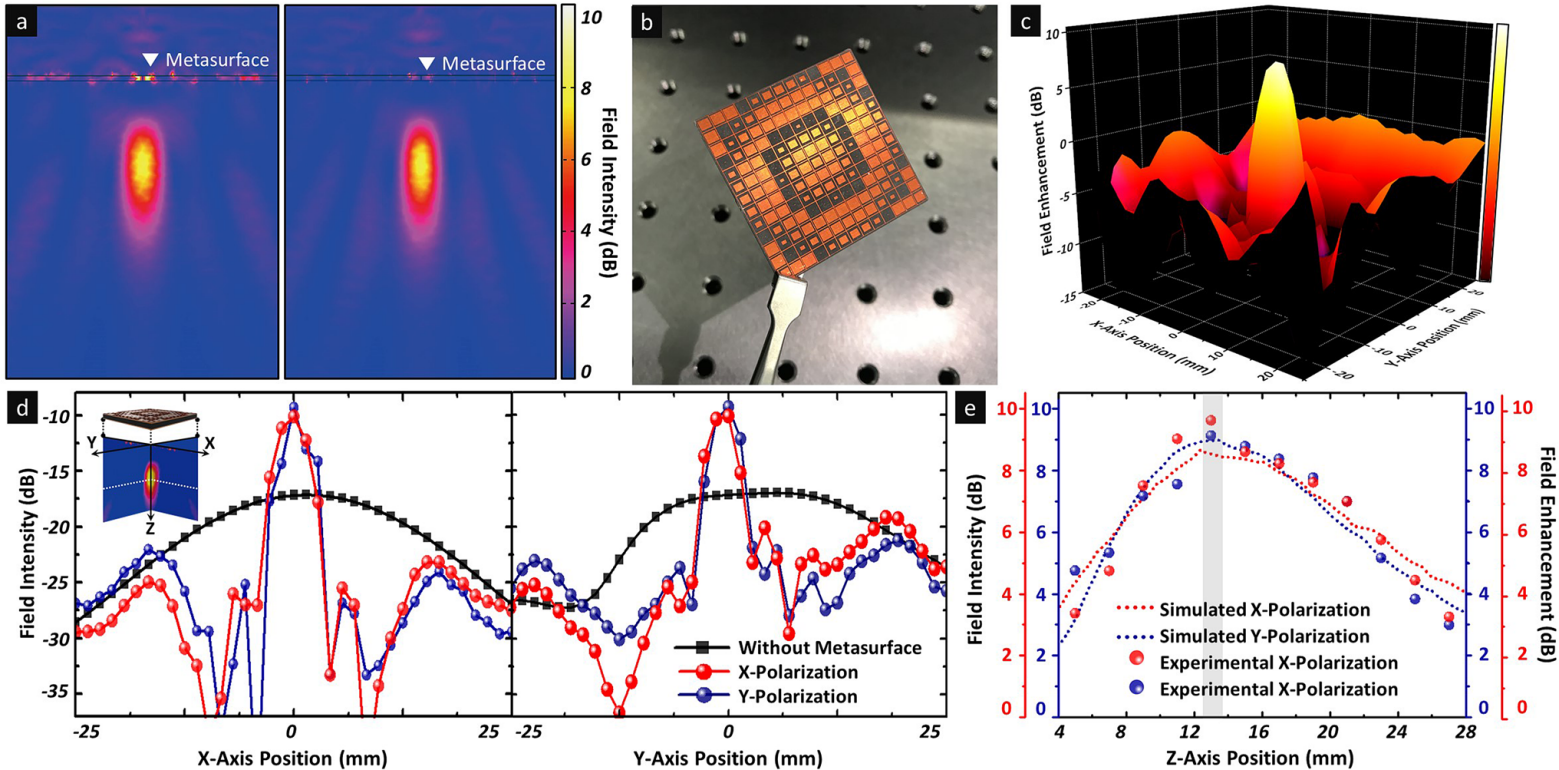
Electromagnetic wave focusing characteristics of proposed metasurface lens at 38 GHz. (**a**) Simulated field intensity distribution of metasurface lens on *xoz*- and *yoz*-planes at 38 GHz. (**b**) Optical image of fabricated metasurface lens. (**c**) Measured three-dimensional electromagnetic focusing profile of proposed metasurface lens. (**d**) Focused field intensity at focal point for x- and y-polarised incident waves. Inset shows the cutplane for verifying the focused intensity at focal point and white dotted line presents the focal plane. (**e**) Simulated and experimental field intensity distributions along the z-axis at 38 GHz for incident x-and y-polarised waves.

Based on the wavefront controllability, to confirm the electromagnetic-wave-focusing effect by the proposed metasurface lens, the field intensity distribution for a normally incident plane wave with linear polarisation was investigated, as shown in Fig. 2a. For a normally incident electromagnetic wave propagating along the z-axis, strongly concentrated electromagnetic fields were generated in both the *xoz*- and *yoz*-planes with identical focal lengths of 13 mm at 38 GHz, with a full-width half-maximum focal spot size of 3.56 mm in the focal plane. Based on the simulated results, the proposed single-layer metasurface lens was fabricated on a 0.787-mm-thick RT/duroid 5880 substrate using conventional photolithography (see Supplementary Information). Figure 2b shows an optical image of the fabricated metasurface lens. A near-field measurement setup was implemented in an anechoic chamber using a linearly polarised horn antenna as a plane wave source and a WR-28 waveguide probe antenna as a detector for scanning the surface area of the metasurface lens at the focal point (see Supplementary Fig. S3a). Figure 2c shows the experimental spatial field enhancement distribution results for the three-dimensional electromagnetic focusing profile of the proposed metasurface lens. As shown in Fig. 2c, a peak field enhancement of 7.84 dB was achieved at the centre of the metasurface lens, whereas the field intensity was suppressed at other locations, implying a strong focusing effect of the proposed structure. Furthermore, for the quantitative analysis of the electromagnetic-wave-focusing characteristics, the two-dimensional field intensity profiles at the focal point were measured, as shown in Fig. 2d. In both the *xoz*- and *yoz*-planes, a focused and enhanced field profile was confirmed at the centre of the metasurface lens, indicating the maximum increased field intensity level of 7.84 dB with a focal spot half-maximum of 3.6 mm in the focal plane, as expected from the simulated result. In addition, we obtained a field enhancement of 7.25 dB for the x-polarised incident wave by the metasurface lens, i.e. a difference of only 0.6 dB compared with that for y-polarisation and can be considered as minimal perturbation from the measurement. Therefore, the proposed metasurface lens realised polarisation independence although it was composed of asymmetrically designed unit cells along the polarisation directions. Moreover, to verify the exact focal length of the metasurface lens ranging from 4 to 28 mm, the field intensity distributions for both polarisation states along the z-axis were analysed. As shown in Fig. 2e, the peak field intensities corresponding to the focal points were observed at z = 13 mm, and the field distribution along the z-axis agreed well with the simulated results for both the x- and y-polarisation states at 38 GHz.

**Figure 3:**
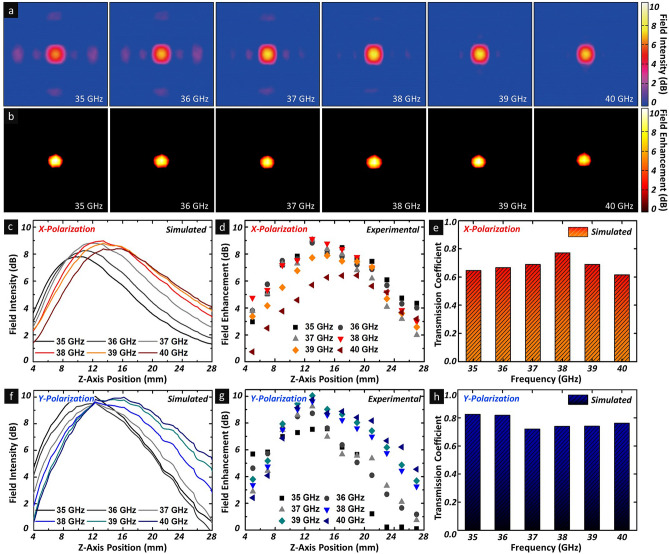
Broadband-focusing characteristics of proposed metasurface lens. (**a**) Simulated field intensity distribution and (**b**) measured field enhancement of metasurface lens on z-plane. (**c**) Simulated and (**d**) experimental field intensity distributions along z-axis ranging from 35 to 40 GHz for x-polarised incident waves. (**f**) Simulated and (**g**) experimental field intensity distributions along z-axis ranging from 35 to 40 GHz for x-polarised incident waves. Simulated transmission coefficient results for (**e**) x- and (**h**) y- polarised incident waves at focal point ranging from the 35 to 40 GHz for y-polarised incident waves.

Generally, it is well known that meta-atoms or metamaterials exhibit narrow bandwidth characteristics or high Q-factors owing to their intrinsically localised surface plasmon resonance properties, resulting in limited operating bandwidth and high insertion loss at off-resonance frequencies that reduce the efficiency of metamaterial-based components. Therefore, several approaches have been proposed to extend the operating frequency bandwidth of metamaterials to realise high-efficiency and ultrathin planar lenses^39,40^. In this regard, the focusing property of the proposed metasurface lens was investigated over a broad bandwidth ranging from 35 to 40 GHz. To demonstrate the electromagnetic-wave-focusing characteristics, the spectral field distributions and intensity profiles were analysed, as shown in Fig. 3. Figure 3a,b shows the simulated field distribution and experimental field enhancement characteristics, respectively, in the focal plane of the metasurface lens at 35, 36, 37, 38, 39, and 40 GHz. Interestingly, as the frequency increased from 35 to 40 GHz, the focal length extended gradually from 9 to 16 mm, as shown in the simulated (Fig. 3c,f) and experimental (Fig. 3d,g) results. Specifically, these results are attributable to the deviated phase shift profiles induced from the parabolic distribution and approximated linear distributions^41^. Although the field distribution results indicated the frequency dependency of the field intensity, the proposed metasurface lens maintained the electromagnetic-wave-focusing characteristics in the targeted frequency range.

Furthermore, the polarisation dependency of the focusing characteristics were analysed, as shown in Fig. 3c–h. In terms of the asymmetrically designed unit cells in the polarisation directions, the frequency dependency of the focused field intensity indicated a slight difference between the x- and y-polarised incident waves, as shown in Figs. 3c,d,f,g respectively. For the x-polarised incident wave, the maximum focused field intensity was observed at 38 GHz, whereas the y-polarised results showed a reverse trend for the operating frequency range. We believe that this phenomenon might have originated from the rectangular shape unit cells that exhibited the opposite transmission coefficient tendency as the operating frequency increased for the x- and y-polarisation states. For the x-polarised incident wave, as shown in Fig. 3e, the transmission coefficient showed a maximum value of 0.772 at 38 GHz, unlike that at 35–40 GHz. Meanwhile, as shown in Fig. 3h, for the y-polarised incident wave, the transmission coefficient showed the opposite tendency, the transmission coefficient showed a minimum transmission coefficient of 0.738 at 38 GHz, unlike that at 35–40 GHz.

**Figure 4:**
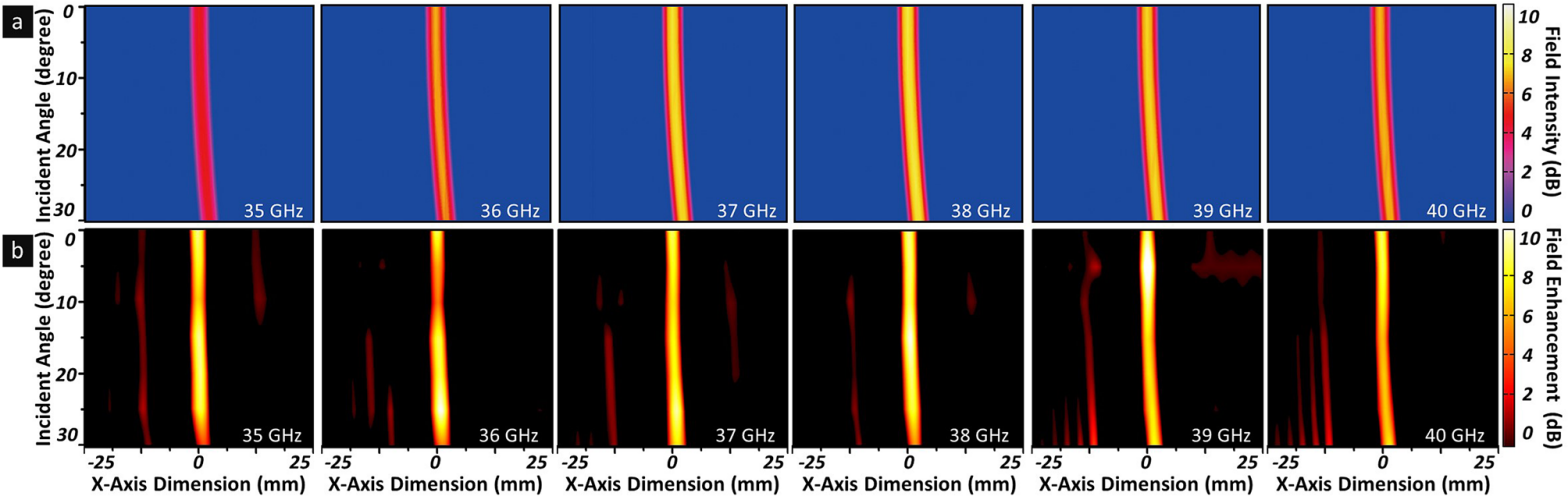
Incident angle independency of proposed metasurface lens. Comparisons of (**a**) simulated and (**b**) experimental results for focal point and focused field intensity distribution with incident angles of 0°, 5°, 10°, 15°, 20°, 25°, and 30° for frequency ranging from 35 to 40 GHz.

Finally, the incident angle behaviour of the proposed metasurface lens was analysed based on the change in the focal points and the focused field intensity profiles as the incident angle was controlled from 0° to 30**°** in the *xoz*-plane for 35–40 GHz, as shown in (see Supplementary Fig. S3b). For the main operating frequency at 38 GHz, as shown in Fig. 4a, in accordance with the increase in the incident angles, the position of the focal point shifted slight compared with the normal incident wave. At an incident angle of 30°, the focal point shifted by 2.7 mm from the origin, i.e. smaller than half of the wavelength ($< 0.34~\lambda$). Furthermore, the focused field intensity was maintained without any fluctuation in the focal length, even at the shifted focal point, despite the oblique incident angles. In particular, the geometrical dimension of the focused beam spot remained constant regardless of the incident angle. Furthermore, to confirm this incident angle independency, focal point measurements were performed over the operating frequencies as the incident angle was controlled, similar to the simulation. As shown in Fig. 4b, at 38 GHz and an incident angle of 30°, the focal point shifted by 3.0 mm from the origin, demonstrating consistency with the simulated result; this was also observed at other operating frequencies from 35 to 40 GHz. Moreover, the size of the focal spots and the field enhancements corresponded well with the simulated results over the operating frequency band. Therefore, the proposed metasurface lens effectively and accurately focused the incident electromagnetic wave at the desired position for wide incident angles without sacrificing focusing properties, thereby providing promise for various practical applications, including communications, wireless power transfer systems, as well as high-gain and high-directional antennas.

## Discussion

A phase gradient single-layer metasurface lens was successfully developed to achieve a fixed focal point of 13 mm regardless of incident angles of incident millimeter waves from 35 to 40 GHz. By controlling the spatial phase distribution on the metasurface lens via arranging low-Q resonant unit cells that exhibit a wide phase modulation range and high transmission coefficient, a subwavelength-thick (< $\lambda /10)$single-layer metasurface lens was realised, which exhibited polarisation independence at the main operating frequency of 38 GHz. In addition, wide-angle performance was confirmed, as shown by the preservation of identical focusing profiles including the focal point, focal length, and focused field intensity for oblique incident angles up to 30°. Finally, the proposed single-layer broadband incident-angle-independent metasurface lens is expected to benefit the development of compact and high-performance flat optical devices suitable for practical applications in millimeter-wave ranges.

## Methods

### Fabrication process of phase gradient single-layer metasurface lens

The phase gradient single-layer metasurface lens was fabricated on 0.787-mm RT/duroid 5880 laminates exhibiting a permittivity of $\varepsilon _{r}$ = 2.2 and a loss tangent of $tan~\delta$ = 0.0009 using conventional photolithography. To form copper patterns on the top and bottom layers, both layers were coated with photoresist and then exposed using their respective photomasks sequentially using an ultroviolet (UV) source for 30 s under vacuum conditions. After UV exposure, the photoresist was developed using a film stripper (PNR-08, Scheme Muse Execute Trading Co., Korea), and the final copper patterns were formed via etching using a copper etchant (Ferric Chloride Anhydrouse Solvent).

## Supplementary Information

Supplementary Information.
