# Supplementary material for: Single-layer phase gradient mmWave metasurface for incident angle independent focusing
Source: Sci Rep. 2021 Jun 16;11:12671. doi: 10.1038/s41598-021-92083-5 (PMC8209207; doi:10.1038/s41598-021-92083-5)
Supplement: Supplementary file 1 — Supplementary Information. [file 41598_2021_92083_MOESM1_ESM.docx]

Supplementary Information

**Single-Layer Phase Gradient mmWave Metasurface for Incident Angle Independent Focusing**

# Wonwoo Lee1, Semin Jo1, Kanghyeok Lee1, Hong Soo Park2, Junhyuk Yang2, Ha Young Hong2, Changkun Park1,2, Sun K. Hong1,2, Hojin Lee1,2,*

1Department of Information Communication Convergence Technology, Soongsil University, Seoul, 0678, Korea

2School of Electronic Engineering, Soongsil University, Seoul, 0678, Korea

*[hojinl@ssu.ac.kr](mailto:hojinl@ssu.ac.kr)

*Correspondence and requests for materials should be addressed to H. L (email: [hojinl@ssu.ac.kr](mailto:hojinl@ssu.ac.kr)).

**Detailed Layout of Electromagnetic-Wave-Focusing Metasurface Lens**


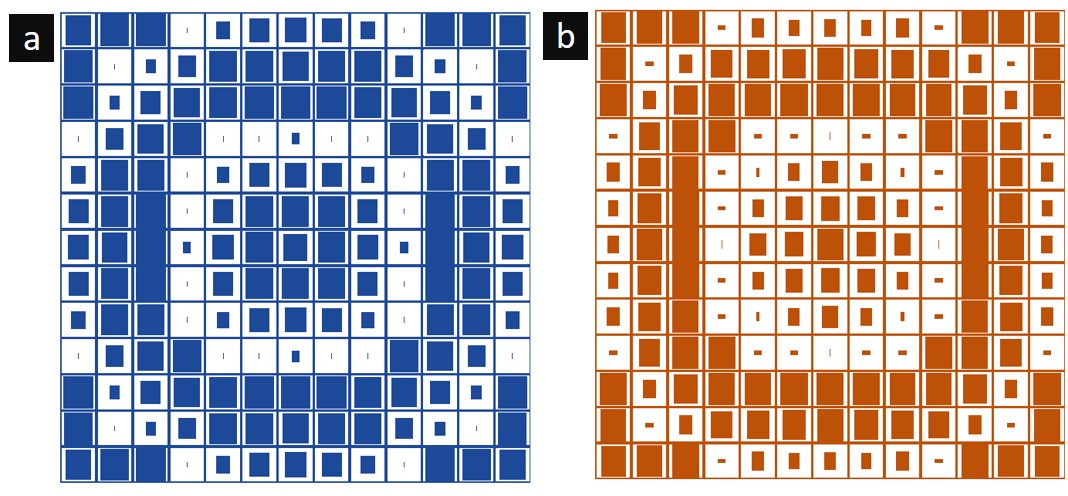


**Figure S1.** Structural layout for (a) top and (b) bottom layers of proposed metasurface.

**
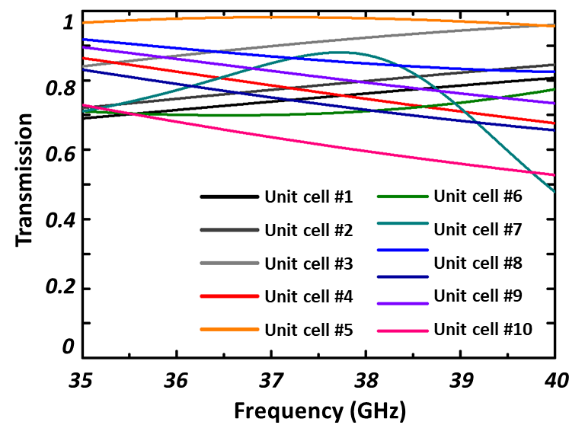
Low Q-Factor Method**

**Figure S2.** Simulated spectral transmission responses for 10 unit cells ranging from 35 to 40 GHz.

In order to realize the broadband electromagnetic wave focusing characteristics ranging from 35 to 40 GHz, the metasurface is required to exhibit low Q-factor resonance property with satisfactory transmissivity at operating frequency range. In this regard, each unit cell in metasurface is also required to have low-Q resonance property, since the metasurface is generated by arranging the unit cells in certain array. Therefore, we optimized the unit cells to have low-Q resonance property by analyzing the spectral transmission responses as shown in Figure R10. Figure R10 shows the spectral transmission responses for the randomly selected 10 unit cells in the metasurface ranging from 35 to 40 GHz, and each transmission spectrum does not show the resonance peak or deep at specific frequency which can be considered as low-Q resonance characteristics the across the operating frequency range. Finally, by arranging the unit cells in accordance with the theoretically determined phase distribution owing to Fermat’s principle, the broadband electromagnetic wave focusing metasurface could be achieved.

**Electromagnetic-Wave-Focusing Metasurface Lens Measurement System**

**
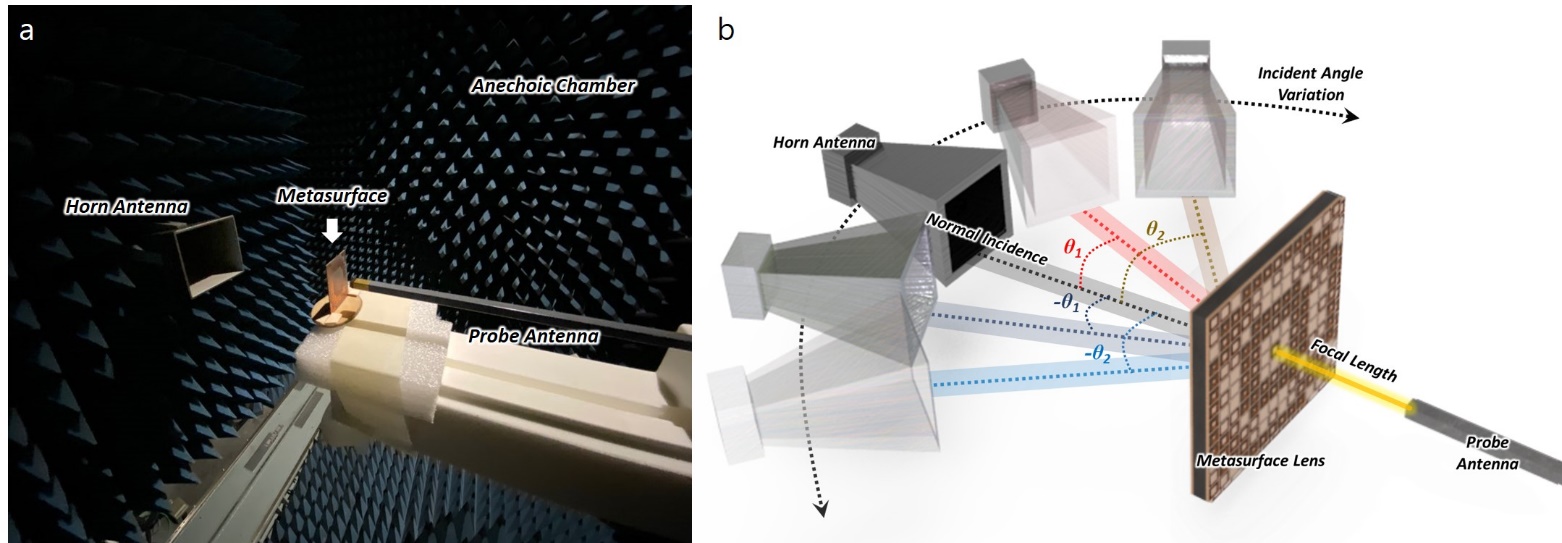
**

**Figure S3**. (a) Anechoic chamber measurement system for electromagnetic-wave-focusing phase gradient single-layer metasurface lens. (b) Conceptual illustration of incident-angle independent measurement system.

Figure S2a shows the measurement system for the electromagnetic-wave-focusing phase gradient single-layer metasurface lens. All measurements were performed in an anechoic chamber that encompassed a frequency range from 2 to 110 GHz to suppress the internal insulation and external noise source. A horn antenna (668A, L3Harris Narda-MITEQ, NY, USA) was adopted as the incident electromagnetic wave source incident, and it was connected to the LO/IF Distribution Unit (Agilent 85309A, CA, USA) to generate electromagnetic waves. For electromagnetic-wave-focusing profile detection, an open ended waveguide probe antenna (WR-28 waveguide, ORBIT/FR, Warminster, PA, USA) was placed at the focal point of the metasurface lens, and the focusing profile was analysed by scanning the metasurface area using a network analyser (Agilent E8364B, CA, USA). Finally, the measurement data were processed and extracted using the ORBIT/FR program (Microwave Vision Group, Israel).

Figure S2b shows a conceptual illustration of the incident-angle-independent measurement system. To tilt the incident angle of the incident electromagnetic wave, the horn antenna was rotated as shown in Figure S1b, maintaining the same distance from the metasurface lens to establish an accurate measurement system. In addition, to minimise the measurement error from the dynamic measurement system, the alignments of the horn antenna, metasurface lens, and probe antenna were optimised for each measurement sequence using a laser aligner.
